# Assessment of carotid artery stenosis and lower limb peripheral ischemia before coronary artery bypass grafting operations: a non-randomized clinical trial

**DOI:** 10.1186/s13019-020-01340-7

**Published:** 2020-09-29

**Authors:** Ihab Ali, Hoda Shokri, Mohammed Abd Al Jawad

**Affiliations:** 1 Department of Cardiothoracic Surgery, Ain Shams University, Cairo, 11355 Egypt; 2 Department of Anesthesiology, Ain Shams University, Cairo, Egypt

**Keywords:** Carotid, Peripheral arterial disease, CABG, Coronary, Endarterectomy

## Abstract

**Background:**

Atherosclerosis is a systemic disease affecting the coronary, carotid, and lower limb arteries. Cerebrovascular accidents and lower limb ischemia are devastating postoperative complications. We aimed to evaluate the role of non-selective routine arterial duplex scanning in patients undergoing coronary artery bypass grafting (CABG).

**Methods:**

This non-randomized clinical trial included 360 patients scheduled for elective isolated CABG who were divided into two groups: low-risk (*n* = 180) and high-risk (*n* = 180). Both groups underwent preoperative carotid and lower limb ultrasound screening for associated arteriopathy.

**Results:**

16 (8.9%) patients and 22 (12.2%) patients showed ≥70% carotid artery stenosis while 11 patients (6.1%) and 20 patients (11.1%) showed ≥50% lower limb arterial stenosis in the low-risk group and the high-risk group, respectively; though the difference was not statistically significant in both the cases (*p* > 0.1).

**Conclusion:**

Routine preoperative peripheral arterial screening by sonography is a feasible and effective strategy to avoid unnecessary post CABG complications.

**Trial registration:**

NCT03516929, Registered in 24 th of April 2018.

## Introduction

Atherosclerosis is widely known as a “systemic” disease that affects many arterial systems in the body. Both coronary and carotid circulations have many similarities like fatty deposits and plaque formation [1]. The coexistence of coronary stenosis, carotid stenosis, and peripheral arterial stenosis is a well-established fact. Age, diabetes, hypertension, and smoking are common risk factors in all arterial disease affections [2].

Stroke is a serious adverse event following coronary artery bypass grafting (CABG) with an incidence of 1.3 to 2.0% and relatively high mortality of up to 38% [3]. It is also associated with increased cardiovascular events, prolonged hospital stay, and expensive health care interventions. Carotid artery stenosis is an important risk factor for cerebrovascular stroke following CABG [3, 4]. The use of non-selective over selective screening for carotid artery stenosis is still debatable [5] with few centers routinely applying it for both high and low-risk patients and most centers reserving it only for those in the high-risk group [6]. Current evidence suggests a 40% reduction in the carotid screening load when reserved for a selected group of patients namely, older patients (> 65 years old) presenting with cerebrovascular disease or carotid bruit without significant impact on the neurological sequel or surgical management [5]. Based on this, both The Society of Thoracic Surgeons (STS) and the American Heart Association (AHA) do not recommend preoperative screening for all patients [7–9].

A significant lower extremity arterial obstruction can lead to ischemic changes in the lower limbs following CABG. It can also lead to delayed wound healing in the legs after saphenous vein graft harvesting. Further, stenosed peripheral lower limb (LL) arteries may cause difficulty in inserting an intra-aortic balloon pump (IABP) whenever needed [10]. Ultrasound duplex screening of both the extracranial and peripheral arterial systems is a simple, noninvasive, cost-effective, and readily available diagnostic modality that can be performed in a clinical setting before the surgery [11–13]. Wanamaker et al. suggest its use non selectively in all CABG patients to reduce postoperative morbidities [14].

The study aimed to evaluate the efficacy of routine carotid and lower extremity arterial screening in asymptomatic patients undergoing CABG.

## Methods

### Study population

This was a prospective, non-randomized, parallel-group study consisting of 360 patients scheduled for elective CABG. All these patients underwent preoperative non-selective carotid and lower limb duplex screening at our institute between April 2018 and May 2020. All patients with ASA physical status II and III undergoing primary, isolated, elective surgical revascularization for triple coronary artery disease were eligible. Morbidly obese patients (BMI ≥ 35), patients with poor left ventricular (LV) functions [ejection fraction (EF) ≤ 40%], previous cardiac operations, and/or previous coronary artery stenting were excluded from the study.

Power Calculations and Sample Size software (PASS® version 15 program; NCSS, LLC, East Kaysville, UT, USA) was used to estimate the sample size. The calculations were based on a previous study [15] which showed that the prevalence of carotid abnormality in low-risk group was 1.2%. Based on a hypothesis of non-inferiority between the low and high-risk group for the prevalence of carotid abnormalities and a 5% margin of non-inferiority between the 2 groups, a sample size of 180 cases per group (360 total) (power of 80%; alpha error at 5%) was needed taking into account a 10% drop out rate.

Patients were divided into two groups, “low-risk group” (*n* = 180) and “high-risk group” (*n* = 180). Based on recommendations by The Society of Thoracic Surgeons (STS), patients were categorized in the high-risk group if they fulfilled at least one of the following criteria: Age ≥ 75 years, patients with a preoperative history of cerebrovascular accident (either stroke or TIA and including transient blindness in one eye, dizziness, confusion, drowsiness, headache, temporary inability to speak or move), patients with carotid bruit, patients with left main stem coronary artery disease, and/or patients with PVD manifested as intermittent claudication, leg pain, leg ulcers, color changes, or loss of hair [7]. Patients with a previous carotid intervention (Endarterectomy CEA or stenting) or lower extremity intervention were also considered high-risk patients.

Written informed consent was obtained from all patients before the start of the study. The study was reviewed and approved by the Institutional Review Board of Faculty of Medicine, Ain Shams University [Ain Shams University Protocol Record (FMASU R 25/2018), ClinicalTrials.gov Identifier: NCT03516929 and was conducted in accordance with the Declaration of Helsinki.

### Patient management

All patients received carotid and LL duplex ultrasound preoperatively which were performed by the same radiologist. The neck examination was performed using a Doppler device (GE Logic S6, USA) together with a linear probe 8.0–12.0 MHz to detect the presence of atheroma or diminished blood flow and included both carotid arteries. For the LL duplex scan, a linear transducer with a variable ultrasound frequency of 9–15 MHz was used, however, a convex transducer with a lower frequency was used for the evaluation of iliac arteries in the pelvic cavity. The procedure was performed with the patient in the supine position. The patient’s hip was abducted and externally rotated with knee flexion to easily visualize the popliteal artery and the posterior tibial artery in the medial calf.

Anesthesia management was standardized to minimize any effect of the anesthetic type on the neurological outcomes. Surgical revascularization was conducted under systemic moderate hypothermia (nasopharyngeal 28 °C–32 °C) during aortic cross-clamping, targeted mean perfusion pressure between 60 and 90 mmHg.

The primary endpoint of the study involved the carotid artery and lower extremity arterial abnormalities confirmed by duplex scan findings. For the carotid arterial system, the degree of stenosis was expressed as the percentage of luminal narrowing estimated by ipsilateral internal common carotid artery flow velocity ratios (duplex ultrasound). Carotid artery stenosis was considered ‘significant’ when there was ≥70% luminal narrowing of the affected internal carotid artery [16]. For lower extremity arterial system, Doppler ultrasound (US) can distinguish between stenosis with a diameter reduction greater than or less than 50% (corresponding to an area reduction of 70%) with a sensitivity of 77 -82% and a specificity of 92 -98% [17, 18]. The US diagnostic criteria for detecting significant stenosis of the lower extremity arteries is a peak systolic velocity ratio of 2.0 as a means of differentiating between stenoses of less than 50% diameter reduction and those of greater than 50% reduction [15, 19]. In our study, we considered peak systolic velocity greater or equal to 2.0 (luminal diameter reduction greater or equal to 50%) as ‘significant’.

The secondary endpoints included the development of new cerebrovascular events and/or distal limb ischemia along with other early 30-day in-hospital outcomes. A cerebral vascular accident or ‘stroke’ was defined as an acute neurological event that occurred within 30 days postoperatively resulting from cerebral circulatory impairment and lasting > 24 h. The outcome of postoperative stroke was defined as the clinical diagnosis of stroke and confirmed by brain imaging [computed tomography (CT), magnetic resonance imaging, or both]. A transient ischemic attack was defined as a temporary neurological deficit attributable to circulatory impairment and lasting < 24 h. Secondary endpoints also included the mortality rate (defined as any death occurring during the same hospital stay), length of hospital stay, ischemic changes, or delayed wound healing of lower limbs.

Different strategies were undertaken during surgery to minimize complications due to PVDs if diagnosed preoperatively: avoid harvesting saphenous vein graft from the ischemic leg or inserting IABP through the stenosed femoral artery, higher bypass perfusion pressure was maintained in case of a stenosed carotid artery, vascular surgeon consultation was sought for any intervention needed, and recommendations for conduit harvesting before admitting the patient for elective surgery.

### Statistical analysis

Data were analyzed using SPSS Statistics version 24.0 (IBM© Corp., Armonk, NY, USA). Normally distributed numerical data are presented as mean and standard deviation. Qualitative data are presented as number and percentage. Normally distributed numerical data were compared using the unpaired *t*-test and categorical data were compared using Fisher’s exact test. *P*-value < 0.05 was considered statistically significant, P-value < 0.001 was considered highly significant.

Logistic regression models were fitted using either a binary logistic regression procedure or a multinomial logistic regression procedure to identify statistically significant predictors of the incidence of cerebrovascular stroke. The logistic regression procedure produces all predictions, residuals, influence statistics, and goodness-of-fit tests using data at the individual case level regardless of how the data are entered and whether or not the number of covariate patterns is smaller than the total number of cases. The multinomial logistic regression procedure internally aggregates cases to form subpopulations with identical covariate patterns for the predictors, producing predictions, residuals, and goodness-of-fit tests based on these subpopulations. If all predictors are categorical or if any continuous predictors take on only a limited number of values so that there are several cases at each distinct covariate pattern, the subpopulation approach can produce valid goodness of fit tests and informative residuals which the individual case level approach cannot.

## Results

The study was reviewed and approved by the Institutional Review Board of Faculty of Medicine, Ain Shams University [Ain Shams University Protocol Record (FMASU R 25/2018), ClinicalTrials.gov Identifier: NCT03516929] and was conducted in accordance with the Declaration of Helsinki. All these patients underwent preoperative non-selective carotid and lower limb duplex screening at our institute between April 2018 and May 2020. A total of 360 patients were assessed for eligibility and all were enrolled in this study with no single case of protocol violation.

**Fig. 1:**
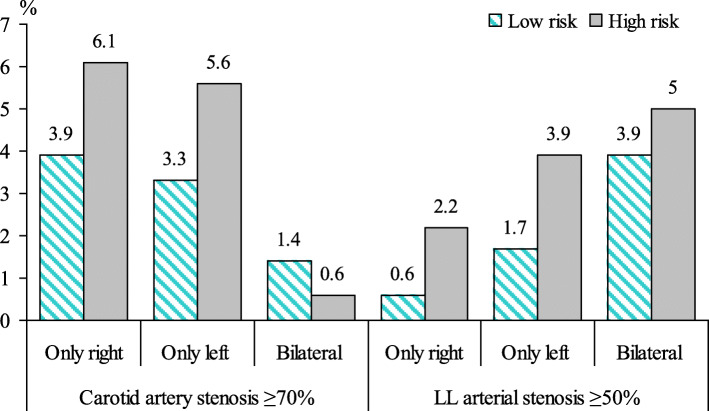
Carotid duplex and LL arterial duplex findings

The demographic parameters (Table 1) indicated that the mean age in the low risk and high-risk group was 48.76 ± 11.22 and 61.06 ± 9.38 years, respectively (*P* < 0.001). The number of smokers in the low-risk group was significantly higher (*P* < 0.001) while the EURO score and the number of patients with age > 75 years were significantly higher in the high-risk group (*P* < 0.001). Table 2 and Fig. 1 shows the carotid duplex and LL arterial duplex findings. The low-risk group showed some patients with accidentally discovered carotid and peripheral arterial lesions. However, there was no statistically significant difference between the two groups in the incidence of carotid artery stenosis, lower limb arterial stenosis. The number of patients needing CT angiography for carotid artery stenosis or CT angiography for LL artery stenosis was comparable between the two groups (*P* = 0.100, *P* = 0.262, respectively). Further, it is worth mentioning that none of the patients in the two groups showed manifestation of total carotid artery occlusion.

**Table 1:** Baseline characteristics of the patients

Parameter		Low-risk group (***N*** = 180)	High-risk group (***N*** = 180)	Tests	
				t/X^**2**^	***P***-value
Age		48.76 ± 11.22	61.06 ± 9.38	11.287	< 0.001**
Sex	Female	26 (14.4%)	35 (19.4%)	1.599	0.206
	Male	154 (85.6%)	145 (80.6%)		
BMI		28.81 ± 2.99	28.65 ± 3.15	0.493	0.622
DM		83 (46.1%)	74 (41.1%)	0.915	0.339
HTN		97 (53.9%)	83 (46.1%)	2.178	0.140
Dyslipidemia		58 (32.2%)	34 (18.9%)	8.410	0.004*
Smoking		44 (24.4%)	21 (11.7%)	9.932	0.002*
EURO score		1.50 ± 0.60	3.49 ± 0.50	34.273	< 0.001**
Age > =75		0 (0.0%)	13 (7.2%)	13.487	< 0.001**
History of PAD		0 (0.0%)	37 (20.6%)	41.238	< 0.001**
History of cerebrovascular events or CVS		0 (0.0%)	26 (14.4%)	28.024	< 0.001**
Presence of carotid bruit		0 (0.0%)	43 (23.9%)	48.833	< 0.001**
Left main stem lesion		0 (0.0%)	70 (38.9%)	86.897	< 0.001**
Preoperative EF		54.79 ± 6.25	54.69 ± 5.75	0.158	0.875

All values are mean ± standard deviation or N (%)

*BMI* Body mass index, *DM* Diabetes mellitus, *HTN* Hypertension, *PAD* Peripheral arterial diseases, *CVS* Cerebrovascular stroke, *EF* Ejection fraction

**Table 2:** Carotid duplex and LL arterial duplex findings and need for CT angiography

		Low risk group N (%)	High risk group N (%)	Chi-square	
				X^**2**^	***P***-value
**Carotid artery stenosis ≥ 70%**	Only right	7 (3.9%)	11 (6.1%)	3.001	0.392
	Only left	6 (3.3%)	10 (5.6%)		
	Bilateral	3 (1.7%)	1 (0.6%)		
**Patients requiring CT angiography for Carotid artery stenosis**		5 (2.8%)	1 (0.6%)	2.712	0.100
**LL arterial stenosis ≥ 50%**	Only right	1 (0.6%)	4 (2.2%)	3.896	0.273
	Only left	3 (1.7%)	7 (3.9%)		
	Bilateral	7 (3.9%)	9 (5.0%)		
**Level of LL arterial affection**	Above knee	2 (1.1%)	5 (2.8%)	3.032	0.220
	Below knee	9 (5.0%)	15 (8.3%)		
**Patients requiring CT angiography for LL artery stenosis**		7 (3.9%)	9 (5.0%)	0.262	0.609

*LL* Lower limb, *CT* Computed tomography

The mean perfusion pressure was significantly higher in the high-risk group (*p* < 0.004) due to targeted higher perfusion pressure in these patients. There was no significant difference in the need for intra-aortic balloon pumping between the two groups (*p* = 0.292). Further, no significant differences were observed in the incidence of distal ischemic changes (*p* = 1.000). The length of ICU stay (*p* = 0.207) and the hospital stay duration (*p* = 0.76) was also similar between the two groups. The incidence of delayed lower limb wound healing (*p* = 1.000) or postoperative neurological complications were also similar between the two groups (*p* = 0.586). No patients required vascular intervention for lower limb arterial stenosis in both the groups (*p* = 1.000), however, 3 patients in the low-risk group and 1 patient in the high-risk group with bilateral ≥70% carotid lesion required carotid endarterectomy (CEA); this did not reach statistical significance (*p* = 0.315) (Table 3).

**Table 3:** Operative data -need for vascular interventions- postoperative data

	Low risk	High risk	Tests	
			t/X^**2**^	***P***-value
ACX time	55.87 ± 8.18	55.77 ± 9.18	0.103	0.918
Total CPB time	90.03 ± 16.71	89.48 ± 16.28	0.313	0.754
Lowest HCT	25.94 ± 1.24	26.06 ± 1.16	0.924	0.356
Lowest temp	30.17 ± 1.80	30.04 ± 1.79	0.676	0.499
Mean perfusion pressure	75.87 ± 5.93	77.89 ± 7.44	2.859	0.004*
Final number of grafts	2.92 ± 0.66	2.93 ± 0.73	0.151	0.880
LIMA alone	170 (94.4%)	165 (91.7%)	1.075	0.300
RIMA alone	3 (1.7%)	6 (3.3%)	1.026	0.311
BIMA	7 (3.9%)	9 (5.0%)	0.262	0.609
Radial (additional)	7 (3.9%)	9 (5.0%)	0.262	0.609
None	0 (0.0%)	0 (0.0%)	0.000	1.000
Combined vascular intervention LL	0 (0.0%)	0 (0.0%)	0.000	1.000
Combined vascular intervention carotid endarterectomy	3 (1.7%)	1 (0.6%)	1.011	0.315
Insertion of IABP	15 (8.3%)	21 (11.7%)	1.111	0.292
Distal ischemic changes	1 (0.6%)	1 (0.6%)	0.000	1.000
ICU stay days	3.46 ± 0.98	3.61 ± 1.02	1.265	0.207
Hospital stay days	7.47 ± 1.8	7.69 ± 1.39	1.356	0.176
Delayed LL wound healing	1 (0.6%)	1 (0.6%)	0.000	1.000
Postoperative neurological complications	6 (3.3%)	8 (4.4%)	0.297	0.586

All values are mean ± standard deviation or N (%)

*ACX* Aortic cross-clamp, *CPB* Cardiopulmonary bypass, *HCT* Hematocrit, *LIMA* Left internal mammary artery, *RIMA* Right internal mammary artery, *BIMA* Bilateral internal mammary artery, *LL* Lower limb, *IABP* Intra-aortic balloon pump, *ICU* Intensive care unit

The majority of patients received left internal mammary artery as their main arterial conduit in both groups without any significant difference (*p* = 0.300). Seven patients in the low-risk group and nine patients in the high-risk group received no saphenous vein graft and were replaced with bilateral internal mammary artery and radial artery conduits due to bilateral LL affection (Table 3).

Logistic regression analysis of patients with > 70% carotid artery stenosis showed that older age (*p* < 0.005) and history of cerebrovascular accidents (*p* = 0.020) were associated with a higher incidence of carotid artery stenosis (*p* = 0.049); however, sex (*p* = 0.762), diabetes (*p* = 0.960), hypertension (*p* = 0.945), dyslipidemia (*p* = 0.992), smoking (*p* = 0.817), history of PAD (*p* = 0.944), history of carotid bruit (*p* = 0.748), presence of main stem lesion (*p* = 0.390), and BMI > 30 (*p* = 0.744) were not associated with increased incidence of carotid artery stenosis (Table 4).

**Table 4:** Results of risk factors analysis for significant carotid stenosis (≥70% luminal narrowing)

Carotid artery stenosis > 70%	Odd ratio	Std. Error	Beta	T-test		95% Confidence Interval	
				t	***P***-value	Lower Bound	Upper Bound
Age	0.008	0.003	0.168	2.830	0.005*	0.002	0.013
Sex	−0.026	0.084	−0.018	−0.304	0.762	−0.191	0.140
DM	−0.007	0.144	− 0.006	− 0.050	0.960	− 0.290	0.275
HTN	−0.007	0.106	−0.007	− 0.069	0.945	− 0.216	0.202
Dyslipidemia	0.002	0.149	0.001	0.010	0.992	−0.292	0.295
smoking	−0.030	0.131	−0.021	−0.231	0.817	−0.287	0.227
History of PAD	0.021	0.293	0.012	0.071	0.944	−0.555	0.597
History of cerebrovascular events or CVS	−0.500	0.215	−0.237	−2.329	0.020*	−0.922	−0.078
Presence of carotid bruit	0.078	0.242	0.046	0.322	0.748	−0.398	0.553
Left main stem lesion	0.127	0.147	0.092	0.862	0.390	−0.163	0.417
BMI	−0.003	0.009	−0.017	−0.327	0.744	−0.022	0.015

Model ANOVA: F = 1.759, *p* = 0.048*

*BMI* Body mass index, *DM* Diabetes mellitus, *HTN* Hypertension, *PAD* Peripheral arterial diseases, *CVS* Cerebrovascular stroke, *EF* Ejection fraction

## Discussion

At present, there are no clear indications whether preoperative carotid screening should be performed in all patients [20].

A meta-analysis by Naylor et al. showed that in both symptomatic and asymptomatic patients with 50–99% stenosis or occlusion and undergoing cardiac surgery, the risk of cardiovascular accidents was 7.4% which increased to 9.1% in patients with 80–99% stenosis. Therefore, it was concluded that patients scheduled for CABG should undergo a duplex scan so that severe carotid stenosis can be diagnosed preoperatively and treated with CEA while patients with moderate carotid stenosis are followed up [21].

A review of existing literature of the North American Symptomatic Carotid Endarterectomy Trial (NASCET), European Carotid Surgery Trial (ECST), and the Asymptomatic Carotid Artery Study (ACAS) concluded that CEA reduces the risk of cardiovascular accidents in both high and low-risk patients [20].

The Society of Thoracic Surgeons and the American College of Cardiology/American Heart Association favors US screening for specific patients (class IIa, level of evidence C) with carotid artery stenosis. The guidelines of the 2012 document “Appropriate Use Criteria for Peripheral Vascular Ultrasound and Physiological Testing” shows all the scenarios of cerebrovascular duplex scan before cardiac surgery. Vascular and Interventional Neurology Society classifies screening of patients undergoing CABG as grade B; however, screening of all patients was considered as grade D [3, 8, 22].

The sensitivity and specificity of a carotid US to diagnose > 70% carotid artery stenosis is 86 -90% and 87 -94%, respectively. However, US has recently been used to detect the degree of carotid artery stenosis. Carotid artery flow velocity and plaque characterization are used to identify patients who are at an increased risk of developing perioperative neurological events after CABG [23, 24].

A study by Kara on the risk of carotid artery stenosis in patients undergoing CABG found that of the eight patients with mean age 48 years diagnosed with ≥70% stenosis, 50% were < 70 years old. If the study had chosen to perform selective carotid DUS only in patients aged ≥70 years, four patients would not have been detected [25]. In our study, 16 patients in the low-risk group were diagnosed with ≥70% stenosis. If we had chosen to perform selective carotid DUS, these patients would have been missed.

A study by Cornily et al. who studied 205 consecutive patients undergoing preoperative carotid screening showed that the prevalence of significant carotid stenosis was 9.1% in the high-risk group and 1.2% in the low-risk group [26]. In our study, the prevalence of carotid artery stenosis was 12.3% in the high-risk group and 8.9% in the low-risk group. Also in his study, the prevalence of stroke was 4.1% in the high-risk group and 0% in the low-risk group. In our study, the prevalence of stroke was 4.4% in the high-risk group and 3.3% in the low-risk group. In the Cornily study, all concomitant or staged carotid endarterectomies/CAB (5/205) and all patients who had perioperative strokes (5/205) were in the high-risk group (*P* = 0.01). In our study, 1 (0.6%) patient in the high-risk group and 3 (1.7%) in the low-risk group required combined intervention (CABG+CEA).

We believe that the association between coronary artery disease and PVD is underestimated in the current guidelines. The non-selective screening will enable us to detect more patients with PVD (either carotid or distal limb diseases). This will carry the following potential benefits:

1. Proper consenting patients with a more accurate estimate of the risk of stroke, limb ischemia, and the feasibility of IABP insertion if needed.
2. The diagnosis of PVD pre-operatively might alter the surgical decision and direct patients towards less invasive approaches.
3. Patients diagnosed with severe carotid disease preoperatively may be offered CEA to reduce the risk of stroke. Perfusionists can be alerted to keep higher mean pressures during bypass to avoid hypoperfusion to the brain.
4. Preoperative diagnosis of severe peripheral limb disease can dictate the site of conduit harvesting to avoid ischemic changes and delayed wound healing. It can also suggest a suitable site for IABP insertion whenever needed.

Being of a single-center experience and a pilot study with a relatively small number of patients, this study was subjected to some limitations: First, patients scheduled for off-pump CABG were not included in the study. Second, we did not estimate if these investigations were cost-effective or not.

## Conclusions

In conclusion, routine preoperative carotid and femoral duplex scans are easy and feasible investigations that can influence the decision making of revascularization and conduit planning. They can help reduce the risk of postoperative complications including cerebrovascular accidents, peripheral limb ischemia, and delayed leg wound healing by performing CEA preoperatively if needed or by adopting bypass strategies to avoid brain hypoperfusion on bypass. LL arterial duplex can also help plan for the most suitable site for IABP insertion whenever needed.

## Data Availability

The data sets used during the current study are available from the corresponding author on reasonable request.

## Abbreviations

CABG: Coronary artery bypass grafting
LL: Lower limb
IABP: Intra-aortic balloon pump
LV: Left ventricular
EF: Ejection fraction
US: Ultrasound
DUS: Doppler ultrasound
CPB: Cardiopulmonary bypass
CVD: Cerebrovascular disease
PVD: Peripheral vascular disease
CT: Computed tomography
CEA: Carotid endarterectomy

## References

[1] Jashari F, Ibrahimi P, Nicoll R, Bajraktari G, Wester P, Henein MY (2013). Coronary and carotid atherosclerosis: similarities and differences. Atherosclerosis..

[2] Seo W-K, Yong HS, Koh S-B, Suh SI, Kim JH, Yu SW (2008). Correlation of coronary artery atherosclerosis with atherosclerosis of the intracranial cerebral artery and the Extracranial carotid artery. Eur Neurol.

[3] Tarakji KG, Sabik JF, Bhudia SK, Batizy LH, Blackstone EH (2011). Temporal onset, risk factors, and outcomes associated with stroke after coronary artery bypass grafting. JAMA..

[4] John R, Choudhri AF, Weinberg AD, Ting W, Rose EA, Smith CR (2000). Multicenter review of preoperative risk factors for stroke after coronary artery bypass grafting. Ann Thorac Surg.

[5] Durand DJ, Perler BA, Roseborough GS, Grega MA, Borowicz LM, Baumgartner WA (2004). Mandatory versus selective preoperative carotid screening: a retrospective analysis. Ann Thorac Surg.

[6] Okur FF, Akpinar MB, Uyar IS, Abacilar AF, Yurtman V, Sahin V (2011). Age groups as a risk factor in isolated coronary and concomitant carotid artery surgery. Turk J Vasc Surg.

[7] The Society of Thoracic Surgeons. Choosing Wisely – Promoting conversations between providers and patients. http://www.choosingwisely.org/clinician-lists/society-thoracic-surgeons-routine-evaluation-of-carotid-artery-disease-prior-to-cardiac-surgery/. Accessed 16 Mar 2018.

[8] Hillis LD, Smith PK, Anderson JL, Bittl JA, Bridges CR, Byrne JG (2011). 2011 ACCF/AHA guideline for coronary artery bypass graft surgery: executive summary: a report of the American College of Cardiology Foundation/American Heart Association task force on practice guidelines developed in collaboration with the American Association for Thoracic Surgery, Society of Cardiovascular Anesthesiologists, and Society of Thoracic Surgeons. J Am Coll Cardiol.

[9] Brott TG, Halperin JL, Abbara S, Bacharach JM, Barr JD, Bush RL (2011). 2011 ASA/ACCF/AHA/AANN/AANS/ACR/ASNR/CNS/SAIP/SCAI/SIR/SNIS/SVM/SVS guideline on the management of patients with extracranial carotid and vertebral artery disease: executive summary: a report of the American College of Cardiology Foundation/ American Heart Association Task Force on Practice Guidelines, and the American Stroke Association, American Association of Neuroscience Nurses, American Association of Neurological Surgeons, American College of Radiology, American Society of Neuroradiology, Congress of Neurological Surgeons, Society of Atherosclerosis Imaging and Prevention, Society for Cardiovascular Angiography and Interventions, Society of Interventional Radiology, Society for Vascular Surgery. J Am Coll Cardiol.

[10] Lau JF, Weinberg MD, Olin JW (2011). Peripheral artery disease. I. Clinical evaluation and noninvasive diagnosis. Nat Rev Cardiol.

[11] Mohler ER, Gornik HL, Gerhard-Herman M, Misra S, Olin JW, Zierler RE (2012). ACCF/ACR/AIUM/ASE/ASN/ICAVL/SCAI/SCCT/SIR/SVM/SVS 2012 appropriate use criteria for peripheral vascular ultrasound and physiological testing part I: arterial ultrasound and physiological testing. J Am Coll Cardiol.

[12] Flanigan DP, Ballard JL, Robinson D, Galliano M, Blecker G, Harward TR (2008). Duplex ultrasound of the superficial femoral artery is a better screening tool than ankle-brachial index to identify at risk patients with lower extremity atherosclerosis. J Vasc Surg.

[13] Mohler ER, Bundens W, Denenberg J, Medenilla E, Hiatt WR, Criqui MH (2012). Progression of asymptomatic peripheral artery disease over 1 year. Vasc Med.

[14] Wanamaker KM, Moraca RJ, Nitzberg D, Magovern GJ (2012). Contemporary incidence and risk factors for carotid artery disease in patients referred for coronary artery bypass surgery. J Cardiothorac Surg.

[15] Ubbink DT, Fidler M, Legemate DA (2001). Interobserver variability in aortoiliac and femoropopliteal duplex scanning. J Vasc Surg.

[16] Eagle KA, Guyton RA, Davidoff R (2004). ACC/AHA 2004 guideline update for coronary artery bypass graft surgery: a report of the American college of cardiology/American heart association task force on practice guidelines (committee to update the 1999 guidelines for coronary artery bypass graft surgery). Circulation..

[17] Zierler RE, Zwiebel WJ, Pellerito JS (2004). Ultrasound assessment of lower extremity arteries. Introduction to vascular ultrasonography.

[18] Shaalan WE, French-Sherry E, Castilla M, Lozanski L, Bassiouny HS (2003). Reliability of common femoral artery hemodynamics in assessing the severity of aortoiliac inflow disease. J Vasc Surg.

[19] Visser K, Hunink MG (2000). Peripheral arterial disease: gadolinium-enhanced MR angiography versus color-guided duplex US—a meta-analysis. Radiology..

[20] Anastasiadis K, Karamitsos TD, Velissaris I, Makrygiannakis K, Kiskinis D (2009). Preoperative screening and management of carotid artery disease in patients undergoing cardiac surgery. Perfusion..

[21] Naylor AR, Bown MJ (2011). Stroke after cardiac surgery and its association with asymptomatic carotid disease: an updated systematic review and meta-analysis. Eur J Vasc Endovasc Surg.

[22] Naylor AR (2010). Managing patients with symptomatic coronary and carotid artery disease. Perspect Vasc Surg Endovasc Ther.

[23] Nicolaides AN, Kakkos SK, Kyriacou E, Griffin M, Sabetai M, Thomas DJ (2010). Asymptomatic internal carotid artery stenosis and cerebrovascular risk stratification. J Vasc Surg.

[24] U.S. Preventive Services Task Force (2007). Screening for carotid artery stenosis: U S. Preventive Services Task Force recommendation statement. Ann Intern Med.

[25] Kara H (2019). Preoperative carotid duplex scanning in patients undergoing coronary artery bypass grafting. Braz J Cardiovasc Surg.

[26] Cornily JC, Le Saux D, Vinsonneau U, Bezon E, Le Ven F, Le Gal G (2011). Assessment of carotid artery stenosis before coronary artery bypass surgery. Is it always necessary?. Arch Cardiovasc Dis.

